# Promotion of nonalcoholic steatohepatitis by RNA N^6^-methyladenosine reader IGF2BP2 in mice

**DOI:** 10.1093/lifemeta/loac006

**Published:** 2022-06-10

**Authors:** Bing Zhou, Yunchen Luo, Nana Ji, Fei Mao, Liping Xiang, Hua Bian, Ming-Hua Zheng, Cheng Hu, Yao Li, Yan Lu

**Affiliations:** Shanghai Diabetes Institute, Shanghai Key Laboratory of Diabetes Mellitus, Shanghai Clinical Centre for Diabetes, Shanghai Jiao Tong University Affiliated Sixth People’s Hospital, Shanghai, China; Key Laboratory of Metabolism and Molecular Medicine of the Ministry of Education, Department of Endocrinology and Metabolism of Zhongshan Hospital, Fudan University, Shanghai, China; Department of Endocrinology and Metabolism, Shanghai General Hospital, Shanghai Jiao Tong University, Shanghai, China; Key Laboratory of Metabolism and Molecular Medicine of the Ministry of Education, Department of Endocrinology and Metabolism of Zhongshan Hospital, Fudan University, Shanghai, China; Department of Endocrinology and Metabolism, Qingpu Branch of Zhongshan Hospital, Fudan University, Wenzhou, China; Key Laboratory of Metabolism and Molecular Medicine of the Ministry of Education, Department of Endocrinology and Metabolism of Zhongshan Hospital, Fudan University, Shanghai, China; Shanghai Diabetes Institute, Shanghai Key Laboratory of Diabetes Mellitus, Shanghai Clinical Centre for Diabetes, Shanghai Jiao Tong University Affiliated Sixth People’s Hospital, Shanghai, China; Key Laboratory of Metabolism and Molecular Medicine of the Ministry of Education, Department of Endocrinology and Metabolism of Zhongshan Hospital, Fudan University, Shanghai, China; NAFLD Research Center, Department of Hepatology, The First Affiliated Hospital of Wenzhou Medical University, Wenzhou, China; Key Laboratory of Diagnosis and Treatment for The Development of Chronic Liver Disease in Zhejiang Province, Wenzhou, China; Shanghai Diabetes Institute, Shanghai Key Laboratory of Diabetes Mellitus, Shanghai Clinical Centre for Diabetes, Shanghai Jiao Tong University Affiliated Sixth People’s Hospital, Shanghai, China; Department of Endocrinology and Metabolism, Fengxian Central Hospital Affiliated to the Southern Medical University, Shanghai, China; Department of Laboratory Animal Science, Shanghai Jiao Tong University School of Medicine, Shanghai, China; Key Laboratory of Metabolism and Molecular Medicine of the Ministry of Education, Department of Endocrinology and Metabolism of Zhongshan Hospital, Fudan University, Shanghai, China; Institute of Metabolism and Regenerative Medicine, Shanghai Jiao Tong University Affiliated Sixth People’s Hospital, Shanghai, China

**Keywords:** nonalcoholic steatohepatitis, m6A reader, hepatic inflammation, IGF2BP2, TAB2

## Abstract

Nonalcoholic steatohepatitis (NASH) has emerged as the major cause of end-stage liver diseases. However, an incomplete understanding of its molecular mechanisms severely dampens the development of pharmacotherapies. In the present study, through systematic screening of genome-wide mRNA expression from three mouse models of hepatic inflammation and fibrosis, we identified IGF2BP2, an N^6^-methyladenosine modification reader, as a key regulator that promotes NASH progression in mice. Adenovirus or adeno-associated virus-mediated overexpression of IGF2BP2 could induce liver steatosis, inflammation, and fibrosis in mice, at least in part, by increasing Tab2 mRNA stability. Besides, hepatic overexpression of IGF2BP2 mimicked gene expression profiles and molecular pathways of human NASH livers. Of potential clinical significance, IGF2BP2 expression is significantly upregulated in the livers of NASH patients. Moreover, knockdown of IGF2BP2 substantially alleviated liver injury, inflammation, and fibrosis in diet-induced NASH mice. Taken together, our findings reveal an important role of IGF2BP2 in NASH, which may provide a new therapeutic target for the treatment of NASH.

## Introduction

Nonalcoholic fatty liver disease (NAFLD) has become the most common form of chronic liver disease worldwide with lifestyle changes [1, 2]. NAFLD, arising from simple steatosis (NAFL), can further progress to nonalcoholic steatohepatitis (NASH), which is characterized by the presence of hepatic inflammation, liver injury, and/or fibrosis [3, 4]. NASH is associated with increased risk of liver-related complications and all-cause mortality [4]. Unfortunately, while NAFL can be ameliorated by dietary interventions, exercise, and weight loss, NASH is usually irreversible [5]. In the past decades, many molecular mechanisms have been identified to explain the development of NASH [6, 7]. Until now, however, there have been no FDA-approved drugs to treat this disease [6, 7].

N^6^-methyladenosine (m6A) methylation is the most abundant epigenetic modification of RNA molecules in eukaryotes [8], which is catalyzed by a methyltransferase complex consisting of METTL3/METTL14/WTAP and removed by m6A eraser proteins like FTO and ALKBH5 [9]. Besides, m6A methylation can be specifically recognized by its reader proteins, including YTHDC family proteins and IGF2BP family proteins [9]. Through regulating mRNA splicing, mRNA stability, or translation efficiency, m6A methylation-related genes play diverse and important roles in many biological processes, such as embryonic development [10], cell proliferation, and tumorigenesis [11]. Interestingly, emerging studies also showed the potential significance of these genes in metabolic regulation, including white and brown adipocyte differentiation [12, 13], pancreatic β-cell proliferation, and insulin secretion [14, 15]. Besides, our recent study showed that the m6A reader Ythdc2 maintains hepatic lipid homeostasis by decreasing the mRNA stability of lipogenic genes [16]. However, whether m6A methylation-related genes can regulate NASH progression remains poorly understood.

In this study, through transcriptome analysis of hepatic gene expression from three mouse models of liver inflammation and injury, we identified IGF2BP2, an m6A reader, as a key factor that drives NASH progression. We further explored the potential role and molecular mechanism of IGF2BP2 in NASH.

## Results

### Transcriptome screening in mouse models with hepatic inflammation and injury

**Figure 1:**
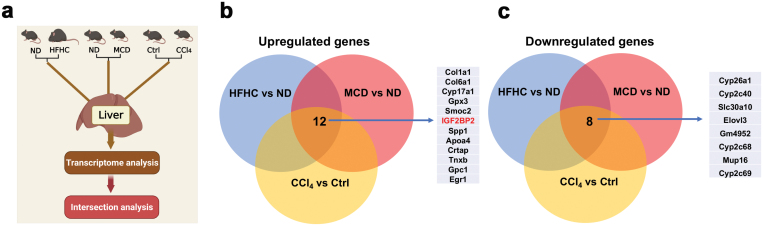
Intersection analysis of dysregulated genes from three mouse models. (a) Experimental design. (b) Venn diagram of the upregulated genes from three groups of mice. (c) Venn diagram of the downregulated genes from three groups of mice.

Compared with NAFL, NASH is typically characterized by persistent hepatic inflammation and hepatocyte death, which in turn activates the wound-healing response to induce liver fibrosis. While many genes have been identified as contributing to NASH severity, their causality remains unclear. To clarify this issue, three mouse models were used in our study (Fig. 1a). In the first model, 8-week-old C57BL/6 male mice were fed a normal chow diet or a high-fat high-cholesterol (HFHC) diet for 28 weeks. Long-term consumption of the HFHC diet has been shown to induce obesity and hepatic steatosis as well as moderate liver inflammation and fibrosis [17, 18]. In the second model, C57BL/6 mice were fed a normal diet or methionine/choline-deficient (MCD) diet for 8 weeks. As expected, consumption of these two diets caused NASH phenotypes in mice, as evidenced by liver pathology as well as increased plasma levels of ALT and AST (Supplementary Figs. S1a–c and S2a–c). In the third model, C57BL/6 mice were chronically treated with vehicle control or carbon tetrachloride (CCl_4_) twice a week for 6 weeks, which can induce severe liver injury and fibrosis. Therefore, the commonalities and differences between these mouse models may help to identify conserved genes that potentially contribute to NASH development.

We then performed global transcriptome analysis using RNA-sequencing (RNA-seq) in the livers from three cohorts (Fig. 1a). Using a *P* value of 0.05 and a fold change greater than 1.5 as cutoff points, we undertook an intersection analysis of three groups of differentially expressed genes (DEGs). As a result, we found that 20 genes were dysregulated in the livers of all mouse models; of which, 12 genes were upregulated and 8 genes were downregulated (Fig. 1b and c). Consistently, abnormal expression levels of Col1a1, Cyp17A1, and Egr1 have been reported to be associated with liver injury and/or fibrosis in previous studies [19–21].

## IGF2BP2 is elevated in three mouse models

**Figure 2:**
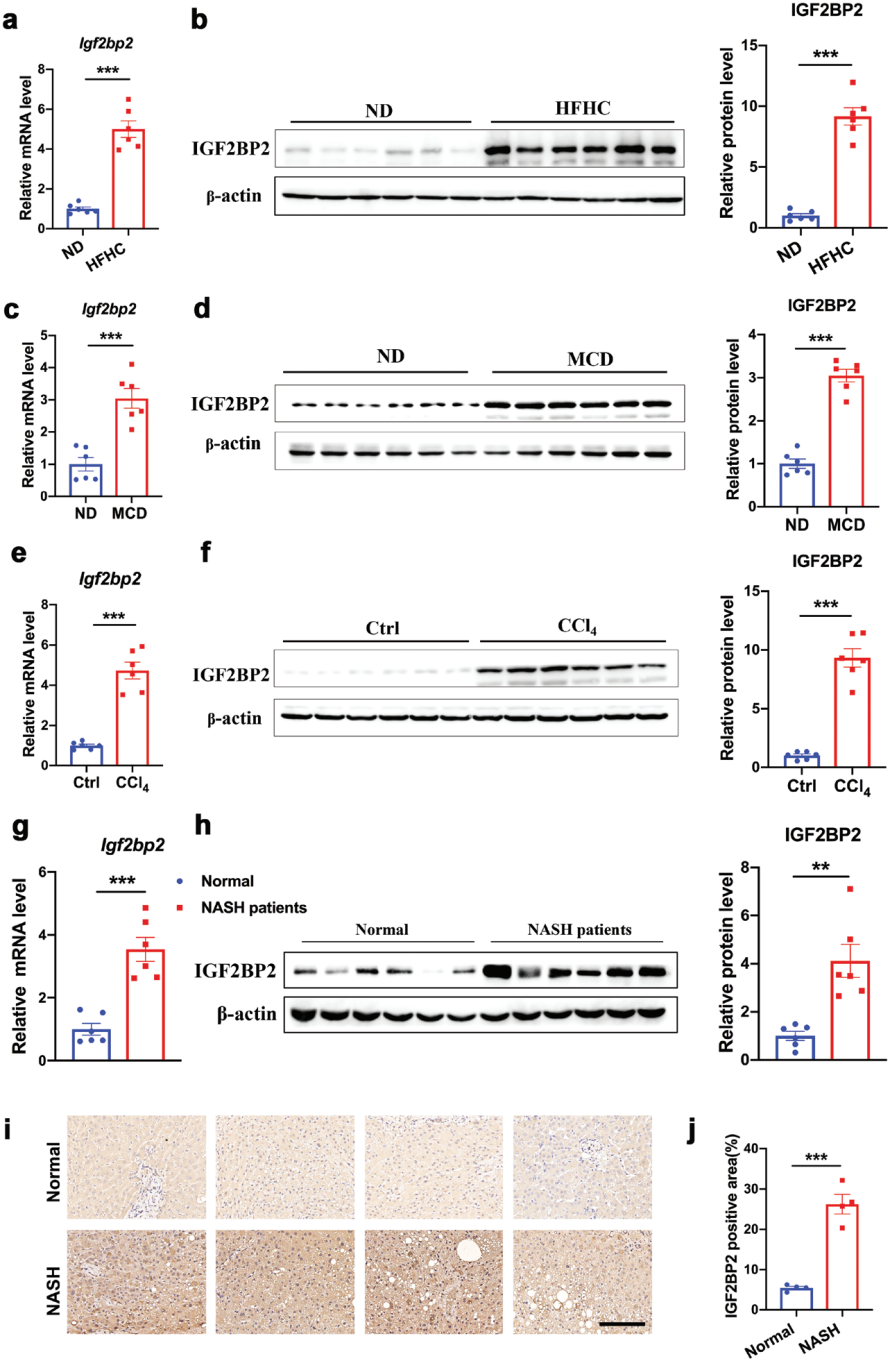
IGF2BP2 is increased in the livers of NASH mice and patients. (a, b) Relative mRNA and protein levels of IGF2BP2 in the livers from HFHC diet-induced NASH mice or control mice. (c, d) Relative mRNA and protein levels of IGF2BP2 in the livers from MCD diet-induced NASH mice or control mice. (e, f) Relative mRNA and protein levels of IGF2BP2 in the livers from CCl_4_- or vehicle control-treated mice. (g, h) Relative mRNA and protein levels of IGF2BP2 in the livers from normal subjects and NASH patients. (i) Representative immunohistochemistry (IHC) staining of IGF2BP2 in liver sections from normal subjects and NASH patients. Scale bar, 50 μm. (j) Quantification of images of IHC staining. *n* = 6 per group (a–h). *n* = 4 per group (i–j). Data are represented as mean ± SEM. **P < 0.01, ***P < 0.001 by two-tailed Student’s *t* test (a–h, j).

Of note, we found a significant upregulation of IGF2BP2 in the livers of three mouse models (Fig. 1b). IGF2BP2 is an m6A reader that can recognize m6A RNA modification and affect the stability and translation of target mRNAs [22]. Considering the important roles of m6A methylation-related genes in liver diseases by previous reports and our recent study [16, 23, 24], IGF2BP2 was chosen for further analysis in this study. Our quantitative real-time PCR (qRT-PCR) and western blots confirmed that mRNA and protein levels of IGF2BP2 were elevated in the livers of HFHC diet-fed, MCD diet-fed, and CCl_4_-treated mice, compared with their matched normal controls (Fig. 2a-f). Furthermore, expression levels of IGF2BP2 were significantly upregulated in the livers of NASH patients, as compared to that in normal subjects (Fig. 2g and h), which was further confirmed by immunochemistry staining (Fig. 2i and j). Collectively, our findings suggest that upregulation of IGF2BP2 in the liver highlights a conserved feature in NASH mice and patients.

## IGF2BP2 deficiency ameliorates hepatic steatosis, inflammation, and fibrosis in NASH mice

**Figure 3:**
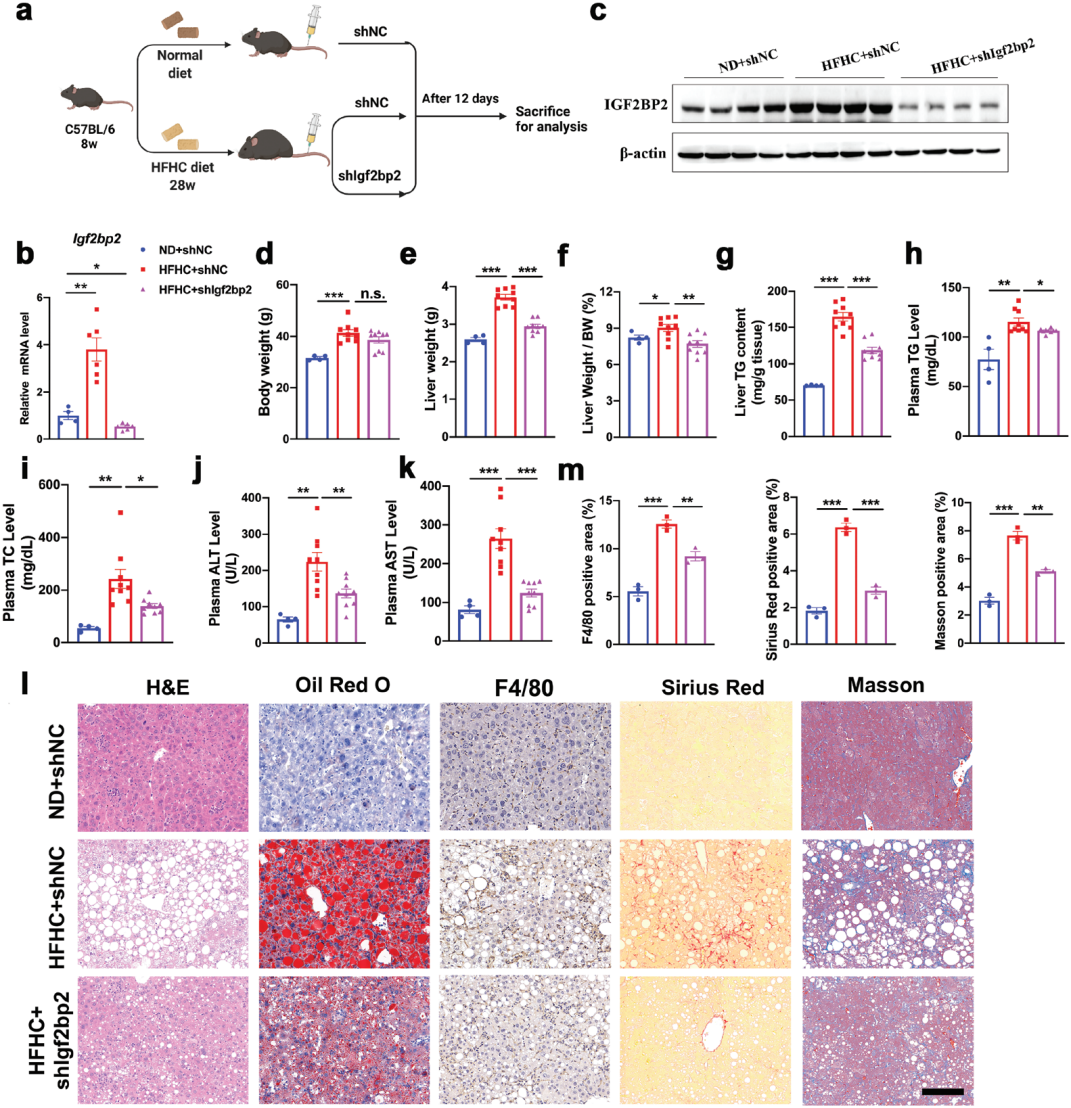
Knockdown of IGF2BP2 improves hepatic steatosis, inflammation, and fibrosis in HFHC diet-induced NASH mice. (a) Experimental design. C57BL/6J mice were fed a normal chow diet or HFHC diet for 28 weeks, starting at 8 weeks of age. Then, mice were administered with adenoviral shRNA targeting Igf2bp2 or negative control for 12 days. *n* = 4–9 per group. (b, c) Relative mRNA and protein levels of IGF2BP2 in the livers of three groups of mice. (d) Body weights. (e) Liver weights. (f) Liver/body weight ratio. (g) Liver triglyceride content. (h) Plasma triglyceride levels. (i) Plasma total cholesterol levels. (j, k) Plasma ALT and AST levels. (l) Liver histology analysis, including H&E, Oil red O, F4/80, Sirius red, and Masson staining. Scale bar, 50 μm. (m) Quantitation of F4/80, Sirius red, and Masson staining. Data are represented as mean ± SEM. *P < 0.05, **P < 0.01, ***P < 0.001 by one-way ANOVA (b, d–k, m).

To test the causality between IGF2BP2 expression and NASH, C57BL/6 mice were fed a normal diet or HFHC diet for 28 weeks and then administered via tail vein with adenoviral shRNA targeting the *Igf2bp2* gene or negative control, respectively (Fig. 3a). Twelve days later, mice were sacrificed for further analysis. This treatment dramatically suppressed IGF2BP2 expression in the livers of HFHC diet-induced NASH mice (Fig. 3b and c). Although body weight was not affected by IGF2BP2 deficiency (Fig. 3d), liver weight and liver/body weight ratio were significantly reduced in the shIgf2bp2-injected mice (Fig. 3e and f). Hepatic and plasma triglyceride contents and plasma total cholesterol levels were also reduced in mice with IGF2BP2 deficiency (Fig. 3g–i). Knockdown of IGF2BP2 also significantly decreased plasma ALT and AST levels (Fig. 3j and k), two markers of liver injury, to an extent similar to normal diet-feeding mice. Histology analysis showed that long-term HFHC diet feeding-associated hepatic steatosis was substantially reduced in IGF2BP2-deficient mouse livers (Fig. 3l). Besides, F/480, Sirius red, and Masson staining indicated that hepatic inflammation and fibrosis were significantly alleviated by Igf2bp2 shRNA treatment (Fig. 3l and m).

We further tested the role of IGF2BP2 deficiency in MCD diet-induced NASH mice. C57BL/6 mice were fed a normal diet or MCD diet for 8 weeks, and then administered with shRNA targeting Igf2bp2 or negative control. In agreement with the observations in the HFHC mice, knockdown of IGF2BP2 also substantially ameliorate hepatic steatosis, liver inflammation, and fibrosis in MCD diet-induced NASH mice (Supplementary Fig. S3a–f). Collectively, these results indicate that IGF2BP2 may exert an important pathogenic role in the occurrence and development of NASH.

## Adenovirus-mediated hepatic overexpression of IGF2BP2 induces NASH phenotypes

**Figure 4.**
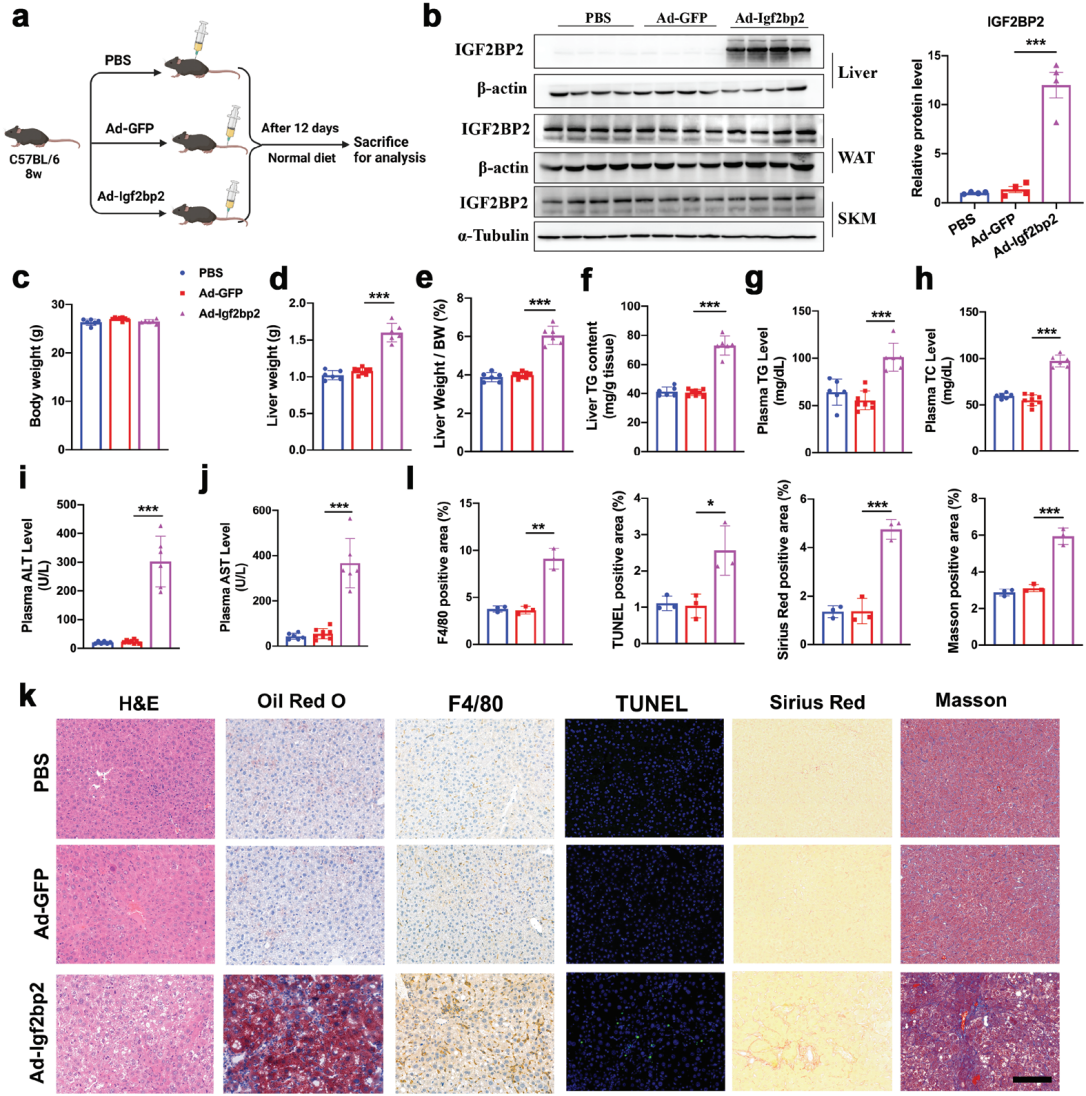
Adenovirus-mediated overexpression of IGF2BP2 induces NASH phenotypes in C57BL/6 mice. (a) Experimental design. C57BL/6J at 8 weeks of age were administered with PBS solution or adenovirus containing IGF2BP2 or GFP. Mice were sacrificed on day 12 for analysis. *n* = 6–8 per group. (b) Protein levels of IGF2BP2 in the liver, white adipose tissue (WAT), and skeletal muscle (SKM) from three groups of mice (left). Protein levels in the liver were quantitated (right). (c) Body weights. (d) Liver weights. (e) Liver/body weight ratio. (f) Liver triglyceride content. (g) Plasma triglyceride levels. (h) Plasma total cholesterol levels. (i, j) Plasma ALT and AST levels. (k) Liver histology and pathology analysis, including H&E, Oil red O, F4/80, TUNEL, Sirius red, and Masson staining. Scale bar, 50 μm. (l) Quantitation of F4/80, TUNEL, Sirius red, and Masson staining. Data are represented as mean ± SEM. *P < 0.05, **P < 0.01, ***P < 0.001 by one-way ANOVA (b–j, l).

We then adopted two approaches to test whether overexpression of IGF2BP2 in the liver can induce NASH pathogenesis. Firstly, C57BL/6 male mice were administered with adenovirus containing *Igf2bp2* (Ad-Igf2bp2) or GFP (Ad-GFP) through tail vein injection. Considering that adenovirus might induce immune response and liver toxicity in mice, an independent group of mice was injected with an equal amount of phosphate-buffered saline (PBS) solution. Three groups of mice were kept on a normal diet-feeding and sacrificed on day 12 post-injection (Fig. 4a). Western blots showed that protein levels of IGF2BP2 were selectively elevated in the livers of the Ad-Igf2bp2 group, compared with the other two groups (Fig. 4b). Besides, its expression in the white adipose tissue and skeletal muscle was not affected by adenovirus infection (Fig. 4b).

While body weights were comparable among the three groups (Fig. 4c), liver weight and liver/body weight ratio was significantly increased in mice with IGF2BP2 overexpression (Fig. 4d and e). Besides, hepatic and plasma triglyceride contents, and plasma total cholesterol levels were elevated in Ad-Igf2bp2-injected mice (Fig. 4f–h). Overexpression of IGF2BP2 also increased plasma ALT and AST levels, indicating severe liver injury (Fig. 4i and j). Histology analysis showed that Ad-Igf2bp2-infected mice developed hepatic steatosis as shown by H&E and Oil Red O staining (Fig. 4k). More importantly, IGF2BP2 overexpression resulted in the increased abundance of F4/80-positive macrophages and TUNEL-positive cells (Fig. 4k and l). Besides, Sirius red and Masson staining showed that liver fibrosis was developed in Ad-Igf2bp2-infected mice (Fig. 4k and l). Collectively, these results suggest that IGF2BP2 overexpression can rapidly induce NASH pathogenesis in mice on a normal chow diet. Of note, all of the metabolic phenotypes examined were comparable between mice administered with PBS and Ad-GFP, suggesting that adenovirus in our study did not induce liver inflammation and injury. In addition, adenovirus-mediated overexpression of IGF2BP2 in mouse primary hepatocytes also resulted in massive cellular triglyceride accumulation and upregulation of inflammatory genes (Supplementary Fig. S4a and b). Collectively, our findings suggest that overexpression of IGF2BP2 can rapidly induce NASH phenotypes in mice in the absence of additional special dietary or chemical interventions.

## Adeno-associated virus-mediated hepatic overexpression of IGF2BP2 induces NASH phenotypes

**Figure 5:**
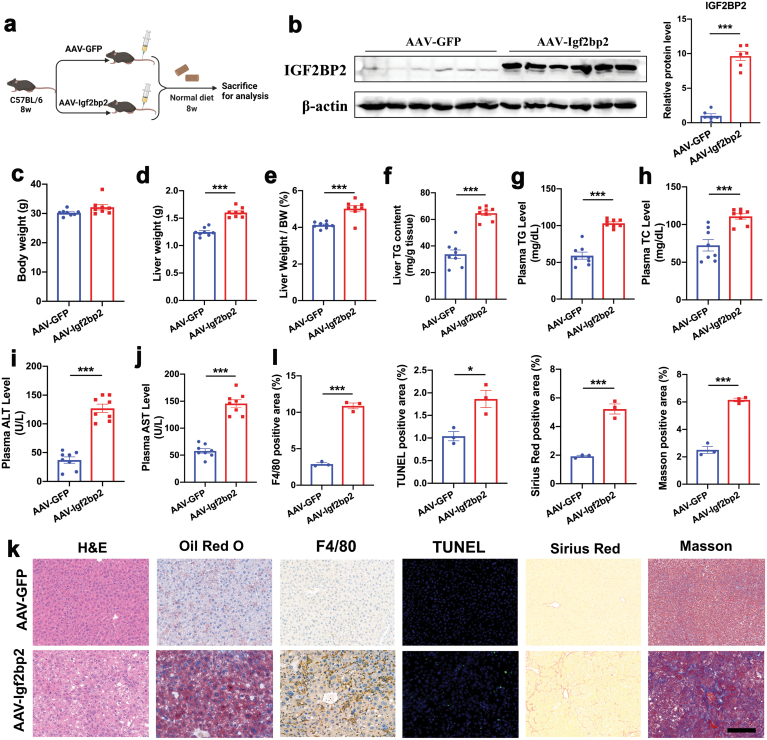
AAV-mediated overexpression of IGF2BP2 induces NASH phenotypes in C57BL/6 mice. (a) Experimental design. C57BL/6J at 8 weeks of age were administered with adeno-associated virus containing Igf2bp2 or GFP. Mice were sacrificed 8 weeks post-injection for analysis. *n* = 8 per group. (b) Protein levels of IGF2BP2 in the livers of two groups of mice. (c) Body weights. (d) Liver weights. (e) Liver/body weight ratio. (f) Liver triglyceride content. (g) Plasma triglyceride levels. (h) Plasma total cholesterol levels. (i–j) Plasma ALT and AST levels. (k) Liver histology analysis, including H&E, Oil red O, F4/80, TUNEL, Sirius red, and Masson staining. Scale bar, 50 μm. (l) Quantitation of F4/80, TUNEL, Sirius red, and Masson staining. Data are represented as mean ± SEM. *P < 0.05, ***P < 0.001 by two-tailed Student’s *t* test (b–j, l).

Considering that adenovirus would gradually be extinguished in the liver, adeno-associated virus (AAV)-mediated gene transduction was used. AAV-mediated gene expression did not induce immune response and could be sustained in the liver for more than 5 months [25, 26]. C57BL/6 mice were administered with AAV9-containing *Igf2bp2* gene or GFP driven by a liver thyroid hormone-binding globulin promoter through tail vein injection. Mice were kept on a normal diet and sacrificed at 8 weeks post-injection (Fig. 5a). Western blots showed that protein levels of IGF2BP2 were elevated in the livers of AAV-Igf2bp2 group, compared with the AAV-GFP group (Fig. 5b). While body weight was comparable between the two groups (Fig. 5c), liver weight and liver/body weight ratio was significantly increased in mice with AAV-Igf2bp2 (Fig. 5d and e). Hepatic and plasma triglyceride contents, and plasma total cholesterol levels were elevated in AAV-Igf2bp2-injected mice (Fig. 5f-h). Persistent overexpression of IGF2BP2 also increased plasma ALT and AST levels in mice (Fig. 5i and j). Histology analysis confirmed that AAV-Igf2bp2-injected mice developed severe hepatic steatosis and inflammation, hepatocyte apoptosis, and liver fibrosis as revealed by H&E, Oil Red O, F4/80, TUNEL, Sirius red and Masson staining, respectively (Fig. 5k and l). Collectively, these results demonstrate that the NASH-inducing effect of IGF2BP2 in mice can be reproduced by AAV-mediated gene transduction.

## Gene expression analysis of IGF2BP2-expressing mice

**Figure 6:**
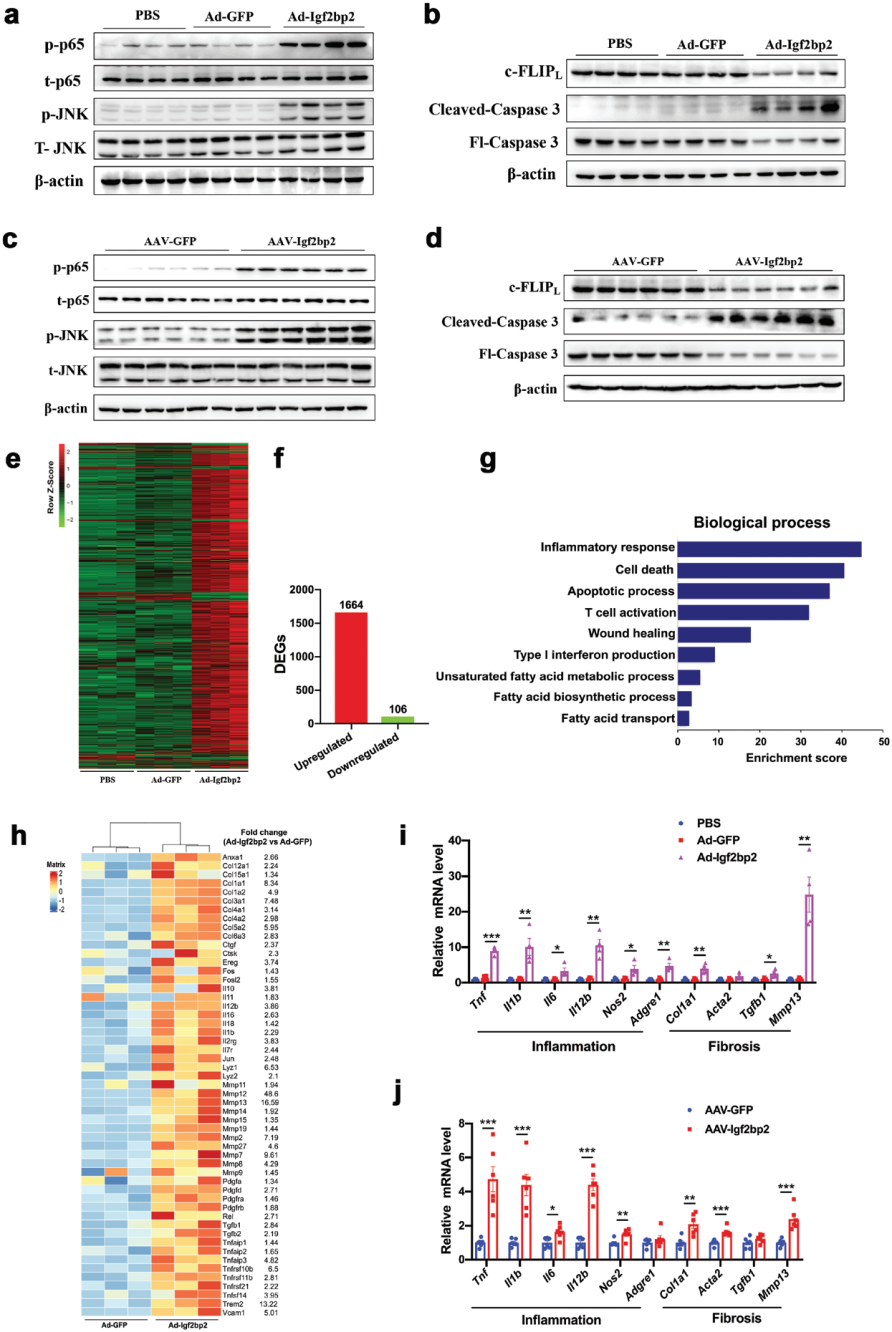
Gene expression analysis of IGF2BP2-overexpressing mice. Gene expression and RNA-seq analysis in the livers from Ad-Igf2bp2-, Ad-GFP-, and PBS-injected mice. (a) Protein levels of phosphorylated p65 and JNK in the livers of three groups of mice. Total p65 and JNK were used as loading controls. p: phosphorylated. t: total. (b) Protein levels of c-FLIP_L_, cleaved, and full-length Caspase 3 in the livers from three groups of mice as indicated. Fl: Full length. (c) Protein levels of phosphorylated p65 and JNK in the livers from AAV-Igf2bp2- and AAV-GFP-injected mice. Total p65 and JNK were used as loading controls. (d) Protein levels of c-FLIP_L_, cleaved and full-length Caspase 3 in the livers from two groups of mice as indicated. (e) Heatmap illustration of gene expression profiles from three groups of mice. (f) Dysregulated genes between Ad-Igf2bp2- and Ad-GFP-injected mice. (g) Gene ontology analysis of upregulated genes. (h) Heatmap illustration showing expression profiles of genes involved in inflammation and fibrosis in Ad-Igf2bp2- and Ad-GFP-injected mice. (i) Relative mRNA levels of genes involved in inflammation and fibrosis in Ad-Igf2bp2-, Ad-GFP- and PBS-injected mice. (j) Relative mRNA levels of genes involved in inflammation and fibrosis in AAV-Igf2bp2- and AAV-GFP-injected mice. Data are represented as mean ± SEM. *P < 0.05, **P < 0.01, ***P < 0.001 by one-way ANOVA (i) or two-tailed Student’s *t*-test (j).

We next examined the molecular features of mice with IGF2BP2 overexpression. First, similar to what occurred in the livers of NASH patients [17], immunoblotting analysis showed that phosphorylation levels of JNK and NF-κB, two major upstream regulators of hepatic inflammation, were increased in the livers of Ad-Igf2bp2-infected mice (Fig. 6a). c-FLIP_L_, a cell death inhibitory protein that suppresses hepatocyte apoptosis [27, 28], is negatively regulated by JNK signaling in the liver [17]. As expected, we found that protein levels of c-FLIP_L_ were reduced in the livers of mice expressing Ad-Igf2bp2 (Fig. 6b). In agreement, protein contents of cleaved Caspase 3 were elevated while full-length Caspase 3 was accumulated (Fig. 6b), which is consistent with increased hepatocyte apoptosis in TUNEL assays (Fig. 4k and l). Alterations of these molecular pathways were also observed in mice expressing AAV-Igf2bp2 (Fig. 6c and d).

We next performed transcriptome analysis by RNA-seq using the livers from Ad-Igf2bp2-, Ad-GFP-, and PBS-injected mice. While the hepatic gene expression pattern was similar between Ad-GFP- and PBS-injected mice, IGF2BP2-overexpressing mice exhibited a strikingly different profile (Fig. 6e). In comparison with Ad-GFP mice, 1664 genes were significantly upregulated and 106 genes were significantly downregulated (Fig. 6f, fold change >2.0, *P* < 0.5). Gene ontology analysis further showed that upregulated genes were mainly enriched in inflammatory response, cell death, T-cell activation, wound healing response, interferon production, and fatty acid metabolic processes, all of which are typical characteristics of NASH livers (Fig. 6g).

We then investigated whether the livers of IGF2BP2-overexpressing mice possessed a similarity in inflammatory and fibrotic gene expression patterns to the livers of NASH patients. In particular, we focused on the expression of genes that have been well established in association with hepatic inflammation and fibrosis [29]. All of these genes were substantially upregulated in the livers of Ad-Igf2bp2-infected mice (Fig. 6h). We further selected some genes to perform qRT-PCR analysis to validate their expression. In support of RNA-seq data, expression levels of candidate genes were significantly upregulated in the livers of mice with IGF2BP2 overexpression (Fig. 6i and j). To further explore to what extent the IGF2BP2-expressing mice could mimic the human NASH pathogenesis in the clinic, a public database in which differential gene expression between clinically pathologically defined NASH patients and normal subjects was analyzed [30]. Notably, expression levels of inflammatory and fibrotic genes in our mouse models were similarly upregulated as observed in NASH patients (Supplementary Fig. S5a and b). Taken together, our findings demonstrate that hepatic overexpression of IGF2BP2 mimicked gene expression profiles of NASH patients.

## IGF2BP2 overexpression induced neutrophil infiltration in the liver

**Figure 7:**
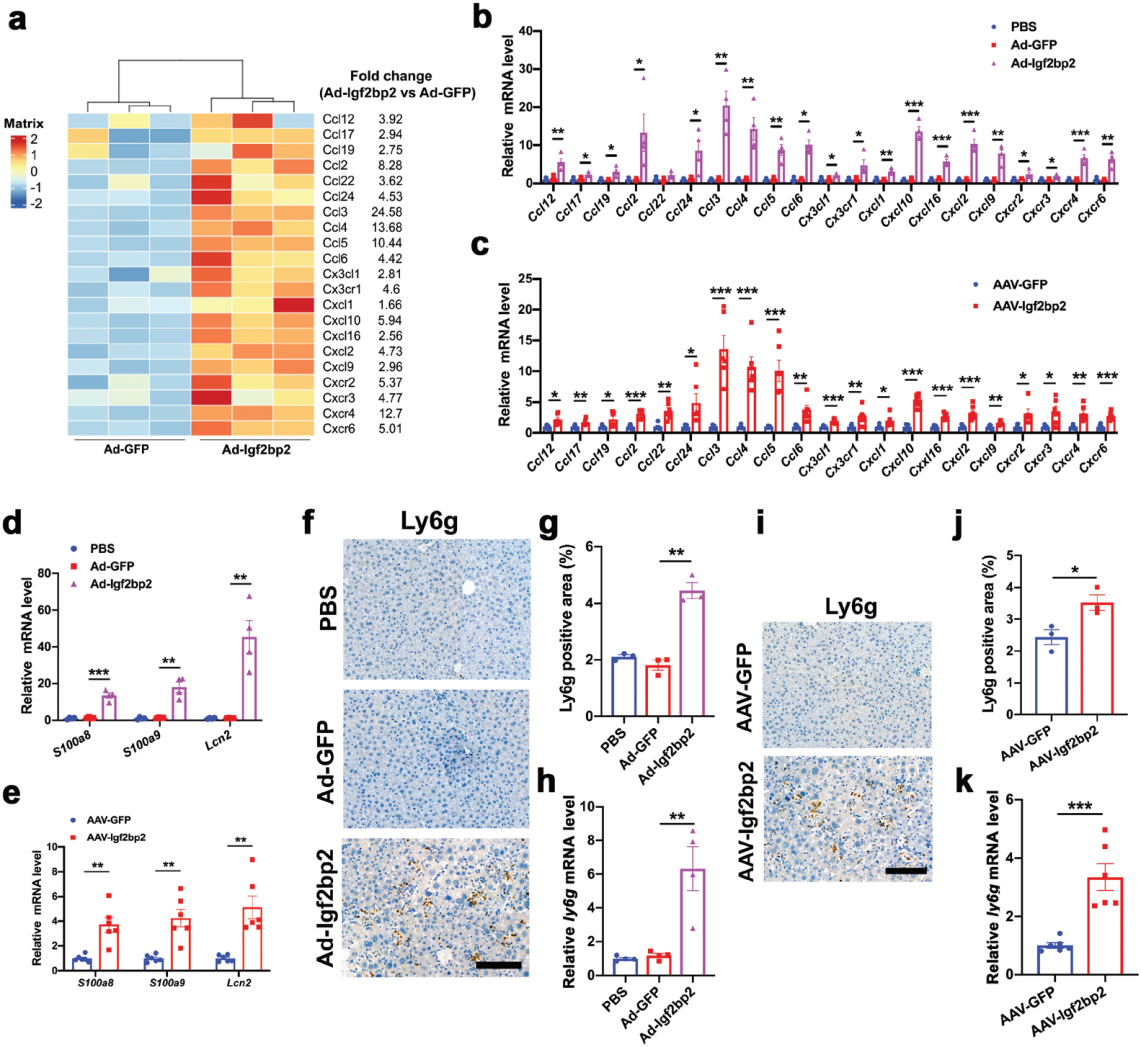
Neutrophil infiltration in the livers of IGF2BP2-overexpression mice. (a) Heatmap illustration shows the expression profiles of chemokines and chemokine receptors in Ad-Igf2bp2- and Ad-GFP-injected mice. (b) Relative mRNA levels of chemokines and chemokine receptors in Ad-Igf2bp2-, Ad-GFP- and PBS-injected mice. (c) Relative mRNA levels of chemokines and chemokine receptors in AAV-Igf2bp2- and AAV-GFP-injected mice. (d) Relative mRNA levels of S100A8, S100A9, and Lcn2 in Ad-Igf2bp2-, Ad-GFP- and PBS-injected mice. (e) Relative mRNA levels of S100A8, S100A9, and Lcn2 in AAV-Igf2bp2- and AAV-GFP-injected mice. (f) Ly6g staining of liver sections from Ad-Igf2bp2-, Ad-GFP-, and PBS-injected mice. (g) Quantification of Ly6g staining. (h) Relative mRNA levels of Ly6g from three groups of mice. (i) Ly6g staining of liver sections from AAV- Igf2bp2- and AAV-GFP-injected mice. (j) Quantification of Ly6g staining. (k) Relative mRNA levels of Ly6g from two groups of mice. Data are represented as mean ± SEM. *P < 0.05, **P < 0.01, ***P < 0.001 by one-way ANOVA (b,d,g,h) or two-tailed Student’s *t*-test (c,e,j,k).

Compared with obese patients with simple steatosis or high-fat-diet-induced fatty liver, another key feature of human NASH is a dramatic infiltration of neutrophils [29, 31]. Neutrophil recruitment is mainly regulated to inflammatory sites by chemokines [32], including hepatocyte-derived Cxcl1 and Cxcl2. Interestingly, expression of these chemokines is regulated by inflammatory signaling, such as NF-κB pathway [33, 34]. As expected, our RNA-seq data showed that mRNA levels of many chemokines and chemokine receptors were elevated in the livers of Ad-Igf2bp2-injected mice (Fig. 7a). Upregulation of these genes was further confirmed by qRT-PCR in the livers of mice expressing Ad-IGF2BP2 or AAV-IGF2BP2 (Fig. 7b and c). Specifically, NF-κB signaling activation and upregulation of Cxcl1 and Cxcl2 were confirmed in mouse primary IGF2BP2-overexpressing hepatocytes (Supplementary Fig. S6a and b). Besides, neutrophil granule proteins [35, 36], including S100 calcium-binding protein A8 (S100A8), S100 calcium-binding protein A9 (S100A9), and lipocalin-2 (Lcn2), were also significantly upregulated in the Ad-Igf2bp2- and AAV-Igf2bp2-infected mice (Fig. 7d and e). Consistently, immunohistochemistry and qRT-PCR analysis showed that Ly6G, a neutrophil marker [37, 38], was increased in the livers of mice with IGF2BP2 overexpression (Fig. 7f–k). Collectively, these results indicate that neutrophil infiltration was increased in the livers of IGF2BP2-overexpressing mice, further supporting the notion that IGF2BP2 can promote NASH progression.

## IGF2BP2 increased the expression and mRNA stability of TAB2

**Figure 8:**
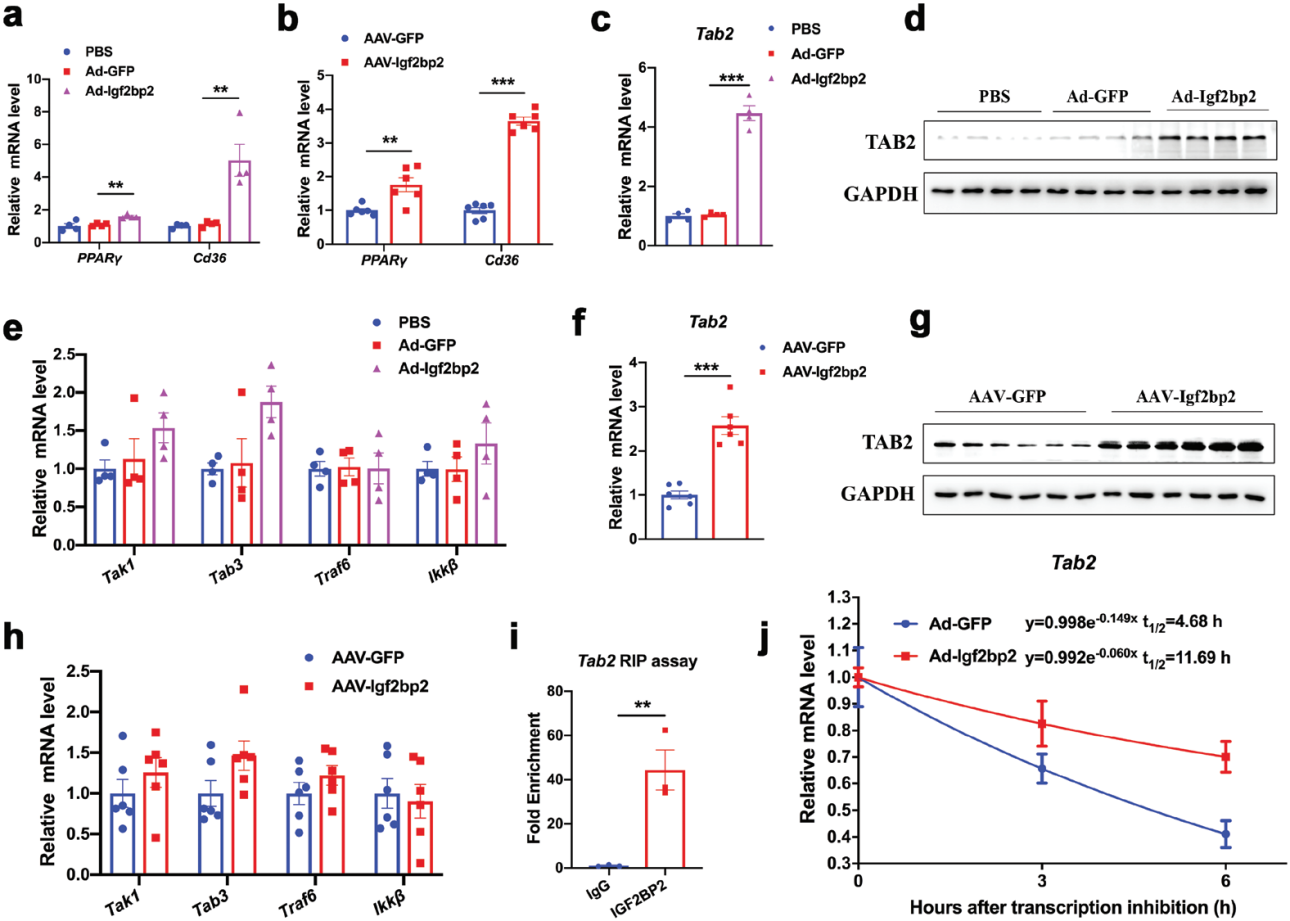
Upregulation of TAB2 by IGF2BP2. (a) Relative mRNA levels of PPARγ and CD36 in the livers of Ad-Igf2bp2-, Ad-GFP-, and PBS-injected mice. (b) Relative mRNA levels of PPARγ and CD36 in the livers of AAV-Igf2bp2- and AAV-GFP-injected mice. (c, d) Relative mRNA levels and protein levels of TAB2 in the livers from Ad-Igf2bp2-, Ad-GFP- and PBS-injected mice. (e) Relative mRNA levels of key regulators of inflammation in the livers of Ad-Igf2bp2-, Ad-GFP- and PBS-injected mice. (f, g) Relative mRNA and protein levels of TAB2 in the livers from AAV-Igf2bp2- and AAV-GFP-injected mice. (h) Relative mRNA levels of key regulators of inflammation in the livers of AAV-Igf2bp2- and AAV-GFP-injected mice. (i) RIP assays showing the binding of IGF2BP2 on the mRNA of Tab2 in HepG2 cells. IgG was used as a negative control. (j) Tab2 mRNA decay in HepG2 cells with IGF2BP2 overexpression versus control. Data are represented as mean ± SEM. **P < 0.01, ***P < 0.001 by one-way ANOVA (a,c,e) or two-tailed Student’s *t*-test (b,f,h,i).

A recent study showed that IGF2BP2 could promote liver steatosis by enhancing PPARγ mRNA stability [39]. In agreement, our results confirmed that mRNA expression levels of PPARγ and its downstream target gene CD36 were increased in the livers of mice expressing Ad- or AAV-Igf2bp2 (Fig. 8a and b). However, studies in humans and mice have shown that activation of PPARγ can ameliorate hepatic inflammation and fibrosis [40–42]. Thus, we speculate that IGF2BP2 may promote steatohepatitis through alternative mechanisms.

Several upstream genes, including Tab2, Tak1, Tab3, Traf6, and IKKβ, have been identified as central signalosomes in the activation of inflammation response [43]. Based on RNA immunoprecipitation (RIP)-coupled high-throughput sequencing [44], hundreds of mRNAs were found to bind with IGF2BP2. By screening this database, we noticed that Tab2 is a potential target of IGF2BP2. Through assembling with its binding partners such as TAK1, TAB2 can activate both JNK and NF-κB pathways [45]. Therefore, we speculated that Tab2 could be a target of IGF2BP2 to contribute to hepatic inflammation. As a result, mRNA and protein levels of TAB2 were dramatically upregulated in the livers of mice expressing Ad-Igf2bp2 (Fig. 8c and d). In contrast, other key regulators of inflammation, including Tak1, Tab3, Traf6, and IKKβ, remained unaffected (Fig. 8e). Similar results were also obtained in the livers of mice expressing AAV-Igf2bp2 (Fig. 8f–h). Our RNA immunoprecipitation assays further showed the interaction of IGF2BP2 with Tab2 mRNA (Fig. 8i), which increased the mRNA stability of Tab2 as revealed in RNA decay analysis (Fig. 8j).

## Discussion

In the present study, we unexpectedly found that IGF2BP2, an m6A reader, was upregulated in the livers from three mouse models of hepatic inflammation, injury, and fibrosis. The m6A modification role of IGF2BP2 is required for the proper regulation of genes involved in embryonic development, tumorigenesis, and hematopoietic stem cell function [22, 46, 47]. Besides, a recent study showed that IGF2BP2 regulates hepatic lipid metabolism through enhancing PPARγ mRNA stability [39]. The steatotic role of IGF2BP2 overexpression was supported by our observations. More importantly, analysis of liver histology showed that, in addition to lipid homeostasis, IGF2BP2 overexpression induced while its knockdown reduced hepatic inflammation, injury, and fibrosis. Consistent with histological analysis, expression levels of genes involved in hepatic inflammation and fibrosis were widely upregulated by IGF2BP2 overexpression at the molecular level. These genes include chemokines (Cxcls, Ccls), proinflammatory cytokines (ILs), collagens (Cols), and matrix metallopeptidases (Mmps). The robust upregulation of chemokines is in accordance with the observation that neutrophil infiltration is increased in the IGF2BP2-overexpressing mice. Recent studies have demonstrated that infiltration of neutrophils around lipotoxic hepatocytes is one of the hallmarks of NASH [29, 31, 32]. Neutrophils have been shown to promote steatosis-to-NASH progression through multiple mechanisms [48], including generation of reactive oxygen species, production of proteases, secretion of pro-inflammatory cytokines, and expression of neutrophil granule proteins. Notably, increased chemokine expression and neutrophil infiltration markers were observed in IGF2BP2-overexpressing mice. Overall, these findings reveal an important role for IGF2BP2 in many aspects of NASH pathogenesis. Since PPARγ activation plays a protective role in NASH pathogenesis, including attenuation of hepatic inflammation, hepatocyte apoptosis, liver injury, and fibrosis [40–42], we speculate that IGF2BP2 may promote steatohepatitis through PPARγ-independent mechanisms. Based on this hypothesis, our results further identified TAB2 as a novel target of IGF2BP2. TAB2 is an upstream central regulator in the inflammation process by activation of both NF-κB and JNK signaling pathways [43]. Indeed, abnormal expression of TAB2 is associated with hepatic steatosis and inflammation in genetically obese mice [49]. However, our findings do not rule out the possibility that, in addition to increasing TAB2 mRNA stability, IGF2BP2 may promote liver inflammation and injury through other mechanisms. Further studies are still needed to identify more targets of IGF2BP2 in NASH progression. Moreover, a recent study reported that IGF2BP2 can promote kidney inflammation and injury through upregulation of TAB3 [50]. Given that TAB2 and TAB3 usually form a complex to activate inflammatory response [45], our results together with this study showed the importance of IGF2BP2 in tissue inflammation and injury.

**Figure 9:**
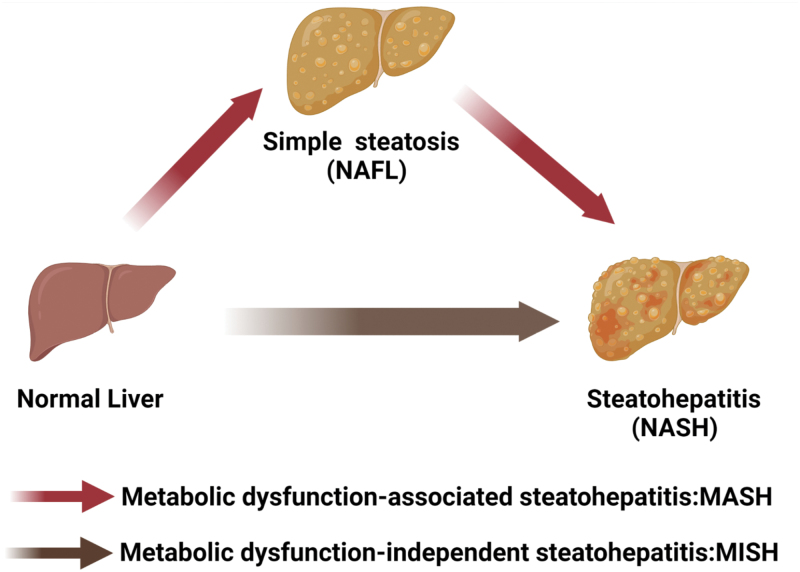
Working model. Nonalcoholic steatohepatitis can be progressed from either simple steatosis or normal liver.

Although our knockdown and overexpression experiments clearly showed the causality between IGF2BP2 expression and NASH development, the reason for the rapid occurrence of NASH by IGF2BP2 overexpression remains unknown. It has been well acknowledged that NASH is usually preceded by NAFL. For instance, our recent study showed that consumption of a 12-week HFHC diet induced simple steatosis in mice without inducing obvious hepatic inflammation and fibrosis, whereas 28 weeks of HFHC diet feeding can cause NASH [51]. We speculate that this chronic process, usually induced by overnutrition, is strongly associated with metabolic dysfunctions, such as obesity and insulin resistance. *M*etabolic dysfunction-*a*ssociated *s*teato*h*epatitis “MASH” was suggested as a more appropriate term to describe it (Fig. 9). However, our current study suggests that NASH could be rapidly and directly converted from a healthy liver by bypassing NAFL. Interestingly, we did not find any changes in body weight, blood glucose levels, and insulin sensitivity during this process (data not shown). This type of *m*etabolic dysfunction-*i*ndependent *s*teato*h*epatitis “MISH” might be caused by specific genetic or epigenetic alterations like IGF2BP2 (Fig. 9).

The lack of translational preclinical animal models has become one of the main obstacles in basic studies and drug discovery in NASH research [52]. Although many dietary-induced, chemical toxin-induced, or genetic mouse models can mimic many of the metabolic changes associated with human NASH, few models can develop hepatic inflammation and fibrosis that properly reflect the pathology and molecular features of NASH patients. Diet-induced models such as long-term consumption of high-fat diets, or genetically induced models such as leptin deficiency (*ob/ob*) and leptin receptor deficiency (*db/db*), can develop hepatic steatosis and insulin resistance. However, it is hard for them to spontaneously progress to steatohepatitis and liver fibrosis [53]. Another common model used in many published literature is MCD diet-feeding mice. This diet can lead to histological appearance similar to NASH in mice; however, the mice usually exhibit severe loss of adipose tissue and liver atrophy. Recent studies have shown that the HFHC diet can induce liver inflammation and fibrosis that resembles human NASH [17, 18]. However, one drawback to the use of the HFHC diet is that it takes too long. In addition to increasing costs, long-term feeding may cause heterogeneity and accelerate premature aging in mice. Besides, the HFHC diet usually contains too much cholesterol (2%), which is super-physiological. Thus, continuous improvement or development of new animal models are still essential for NASH research [52]. Interestingly, our results demonstrated that IGF2BP2-overexpressing mice resemble the gene expression and molecular features of NASH patients. Therefore, adenovirus and AAV-mediated gene transduction of IGF2BP2, or the construction of transgenic mice that stably express IGF2BP2 in hepatocytes, might be a novel way to construct a NASH model. Of note, the NASH-inducing effects of IGF2BP2 did not require any special dietary or chemical interventions. Thus, compared with other reported NASH murine models, one of the main advantages of this model is that it is fast and simple.

In summary, our study revealed that IGF2BP2 facilitates NASH progression, at least in part, through upregulation of TAB2 and activation of hepatic inflammatory response. Of pathophysiological significance, hepatic IGF2BP2 expression is upregulated in NASH mice and patients, suggesting that suppression of IGF2BP2 may serve as a promising therapeutic target for treating this disease.

## Materials and methods

### Animal experiments

C57BL/6J male mice were purchased from the Shanghai Laboratory Animal Company (SLAC, Shanghai, China). For HFHC diet feeding, mice were fed a diet containing 40% of fat, 22% of fructose, and 2% of cholesterol (D09100310, Research Diets Inc.) for 28 weeks. For MCD diet feeding, mice were fed with an MCD diet (A02082002B, Research Diets Inc.) for 8 weeks. For CCl_4_ treatment, mice were intraperitoneally administered with CCl_4_ (1 ml/kg, 1:4 v/v in corn oil) twice a week for 6 weeks. All animal protocols were reviewed and approved by the Animal Care Committees of Shanghai Jiao Tong University School of Medicine and Zhongshan Hospital.

## Human specimens

Liver specimens from healthy controls or biopsy-proven NASH patients were obtained from the Department of Endocrinology and Metabolism at Zhongshan Hospital, Fudan University, China. The exclusion criteria and associated information in detail have been described in recent studies [54, 55]. The human study was approved by the Human Research Ethics Committee of Zhongshan Hospital and conducted in accordance with the 1975 Declaration of Helsinki. Written informed consent was obtained from each subject.

## Adenovirus and adeno-associated virus

Recombinant adenovirus (Adv) for overexpression of *Igf2bp2* gene (NM_183029) was constructed using GV314 vector (GeneChem, Shanghai, China). GFP gene was used as a negative control. Adeno-associated virus (AAV9) was constructed using GV625 vector (GeneChem), driven by a liver-specific thyroxine-binding globulin promoter. Adenovirus-delivered short hairpin RNA (shRNA) was constructed using GV119 vector (GeneChem). For overexpression or knockdown of IGF2BP2, 1 × 10^9^ pfu of purified Adv, 1 × 10^11^ pfu of purified AAV, or 1 × 10^9^ pfu of Adv-shRNA was injected into mice by tail vein injection. The Igf2bp2 shRNA had the following target sequences: 5ʹ-GGAGCAAGTCAACACAGAT-3ʹ. The scrambled shRNA had the following target sequences: 5ʹ-TTCTCCGAACGTGTCACGT-3ʹ.

## Triglyceride and cholesterol measurements

Liver tissues were harvested and homogenized in 5% NP-40 solution and heated up to 100 °C and then cooled down to room temperature. The tissue homogenates were centrifuged and the supernatants were processed for measuring triglyceride and cholesterol contents using commercial kits (catalog K622 and K603, BioVision, Milpitas, USA).

## Histology analysis, F4/80 staining, TUNEL, and Sirius red staining

For H&E staining, liver tissues were fixed in 10% neutral-buffered formalin, embedded in paraffin, and cut into 5 μm sections. Paraffin-embedded tissue sections were subjected to de-paraffinization and rehydration and then were immersed in 95 °C antigen retrieval buffer (10 mM sodium citrate, 0.05% Tween 20, pH 6.0) for 30 min. F4/80 (catalog ab6640, Abcam) and Ly6g staining (catalog 25377, Abcam) were conducted on liver paraffin sections to analyze macrophage accumulation and neutrophil infiltration. Hepatocyte death was determined by TUNEL staining of liver sections with TUNEL Assay Kit (catalog ab206386, Abcam) according to the manufacturer’s instructions. Nuclei were stained with DAPI. Immunostaining was visualized with CaseViewer. For Sirius Red staining, paraformaldehyde-fixed samples were embedded in paraffin, cut into 5-μm-thick sections, and stained with Sirius Red (saturated picric acid containing 0.1% DirectRed 80 and 0.1% FastGreen FCF; Sigma-Aldrich) according to standard procedures. Images were assessed by quantitating histological collagen staining with ImageJ software, taking care to avoid major vessels, and liver capsule.

## RNA sequencing

RNA high-throughput sequencing was performed by Cloud-Seq Biotech (Shanghai, China). Briefly, total RNA was used for removing the rRNAs with the NEBNext rRNA Depletion Kit (New England Biolabs, Inc., MA, USA) following the manufacturer’s instructions. RNA libraries were constructed by using the NEBNext® Ultra™ II Directional RNA Library Prep Kit (New England Biolabs, Inc.) according to the manufacturer’s instructions. Libraries were controlled for quality and quantified using a BioAnalyzer 2100 system (Agilent Technologies, Inc., USA). Library sequencing was performed on an Illumina Hiseq instrument with 150 bp paired-end reads. Paired-end reads were harvested from the Illumina HiSeq 4000 sequencer and were quality controlled by Q30. After 3ʹ adaptor-trimming and low-quality reads removed by cutadapt software (v1.9.3), the high-quality clean reads were aligned to the reference genome (UCSC mm10) with hisat2 software (v2.0.4). Then, guided by the Ensembl gtf gene annotation file, cuffdiff software was used to get the gene-level FPKM as the expression profiles of mRNA, and fold change and *P* value were calculated based on FPKM, and differentially expressed mRNA were identified.

## RNA isolation and quantitative real-time PCR

Total RNAs were isolated from cell lysates or mouse liver tissues using the standard TRIzol method according to the manufacturer’s instructions (Invitrogen). First-strand cDNA was synthesized from each RNA sample using the Reverse Transcription System (Promega). Oligo(dT) was used to prime cDNA synthesis. To analyze gene expression, qRT-PCR was performed using an SYBR Green Premix Ex Taq (Takara Biotechnology) on a LightCycler 480 (Roche, Basel, Switzerland). Relative quantification of gene expression data was done according to the $2^{-\Delta \Delta C_{t}}$ method. The results of relative expression were normalized to mRNA levels of the housekeeping gene Rplp0. The primer sequences for qRT-PCR are available upon request.

## Western blots

Proteins from tissues were harvested using radioimmunoprecipitation buffer containing Tris–HCl (50 mmol/l), NaCl (150 mmol/l), MgCl_2_ (5 mmol/l), EDTA (2 mmol/l), NaF (1 mmol/l), 1% NP40, and 0.1% SDS. The protein concentrations were quantified using commercial kits from Thermo Fisher Scientific. All protein samples were equally subjected to 10% SDS-polyacrylamide gels, transferred to polyvinylidene difluoride membranes by electrophoresis, incubated with primary and secondary antibodies, and finally visualized by a chemiluminescence detection kit (Millipore). The following primary antibodies were used: anti-phospho-P65 at 1:1000 (catalog 3033, Cell Signaling Technology), anti-total P65 at 1:1000 (catalog 8242, Cell Signaling Technology), anti-phospho-JNK at 1:1000 (catalog 9251, Cell Signaling Technology), anti-total JNK at 1:1000 (catalog 9252, Cell Signaling Technology), anti-c-FLIP at 1:500 (catalog sc-5276, Santa Cruz), anti-Caspase 3 at 1:1000 (catalog 9661, Cell Signaling Technology), anti-IGF2BP2 at 1:1000 (catalog 11601-1-AP, Proteintech Group), anti-TAB2 at 1:1000 (catalog 14410-1-AP, Proteintech Group), anti-α-tubulin at 1:5000 (catalog T-6199, Sigma), anti-β-actin antibody at 1:2000 (catalog sc-47778, Santa Cruz), and anti-GAPDH antibody at 1:2000 (catalog sc-32233, Santa Cruz). Quantitation of immunoblots was assessed by ImageJ software.

## RNA immunoprecipitation

RIP experiments were performed using a Magna RIP RNA-Binding Protein Immunoprecipitation Kit (Millipore, Bedford, MA, USA) according to the manufacturer’s instructions. Briefly, harvested HepG2 cells were lysed with RIP lysis buffer on ice and then incubated with anti-IGF2BP2 antibody or control IgG antibody at 4 °C overnight. The immunoprecipitated RNAs were isolated by TRIzol reagent (Invitrogen, CA, USA) and analyzed by qRT-PCR.

## mRNA lifetime

TAB2 mRNA stability was determined in HepG2 cells transfected with Ad-Igf2bp2 or Ad-GFP for 12 h and then treated with actinomycin D (5 μg/ml) (Sigma-Aldrich, Saint Louis, USA) for 6 h, 3 h, and 0 h, respectively. Then, the total RNAs were isolated by TRIzol and expression levels of transcripts of interest were detected by qRT-PCR. With the treatment of actinomycin D, the mRNA transcription was turned off and the degradation rate of mRNA (*K*_decay_) was estimated by the following equation: ln (*C*/*C*_0_) = −*K*_decay_*t*, where *t* is transcription inhibition time (in h), while *C* and *C*_0_ represent mRNA quantity at time *t* and 0. The degradation of Tab2 mRNA followed a first-order kinetics and the half-life was calculated by *t*_1/2_ = ln_2_/*K*_decay_, whereas the *K*_decay_ values were extracted from the exponential trendlines (line of best fit).

## Statistics

All statistical analysis was performed using GraphPad Prism Software (version 8, GraphPad, USA). Data are presented as mean ± SEM. For animal and cellular experiments, a two-tailed unpaired Student’s *t* test was performed to compare between two groups. One-way ANOVA followed by the Student–Newman–Keuls test was used to compare more than two groups. Two-side *P* < 0.05 was considered statistically significant. Statistical significance is displayed as *P< 0.05, **P< 0.01, or ***P< 0.001.

## Supplementary Material

loac006_suppl_Supplementary_Figure

## Data Availability

Any information required to re-analyze the data reported in this study is available from the Corresponding author (Y Lu) upon request.
