# Supplementary material for: Promotion of nonalcoholic steatohepatitis by RNA N6-methyladenosine reader IGF2BP2 in mice
Source: Life Metab. 2022 Jun 10;1(2):161–74. doi: 10.1093/lifemeta/loac006 (PMC11749640; doi:10.1093/lifemeta/loac006)
Supplement: loac006_suppl_Supplementary_Figure [file loac006_suppl_Supplementary_Figure.pdf]

Supplementary Information

Supplementary Figures S1-S6.

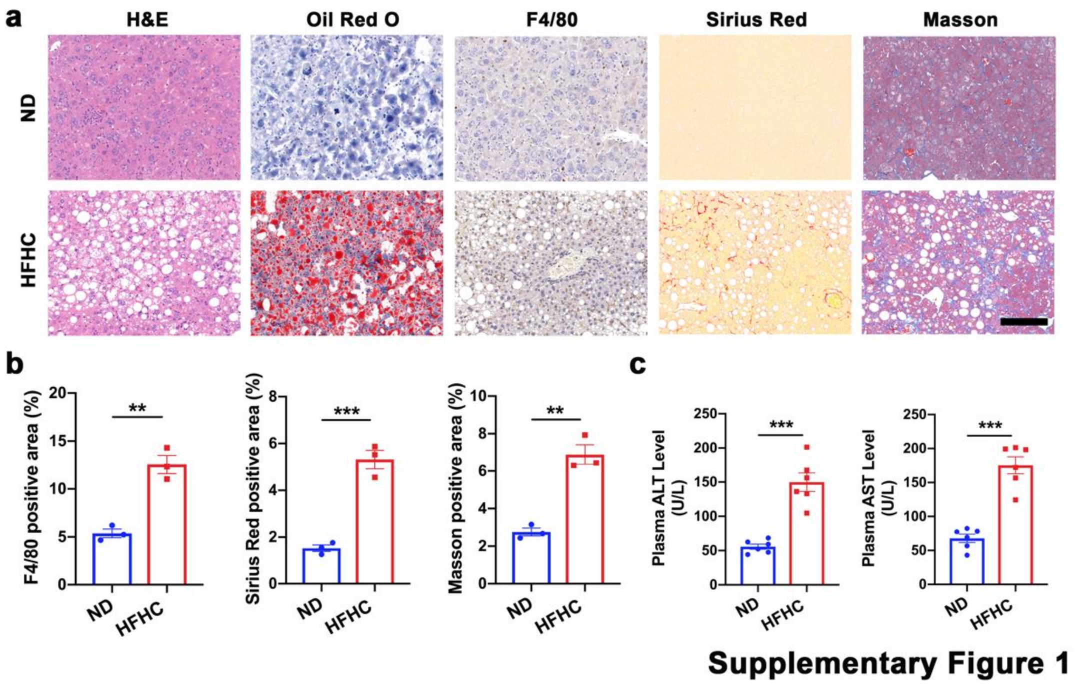

Supplementary Figure S1. Construction of HFHC diet-induced NASH mice. **(a-c)** 8-week-old C57BL/6 male mice were fed a normal chow diet or HFHC diet for 28 weeks. n=6 per group. **(a)** Liver histology analysis, including H&E, Oil red O, F4/80, Sirius red and Masson staining. Scale bar, 50 µm. **(b)** Quantitation of images. **(c)** Plasma ALT and AST levels between two groups of mice. Data are represented as mean ± SEM. ** *P* <0.01, *** *P* < 0.001 by 2-tailed Student's t test (b-c).

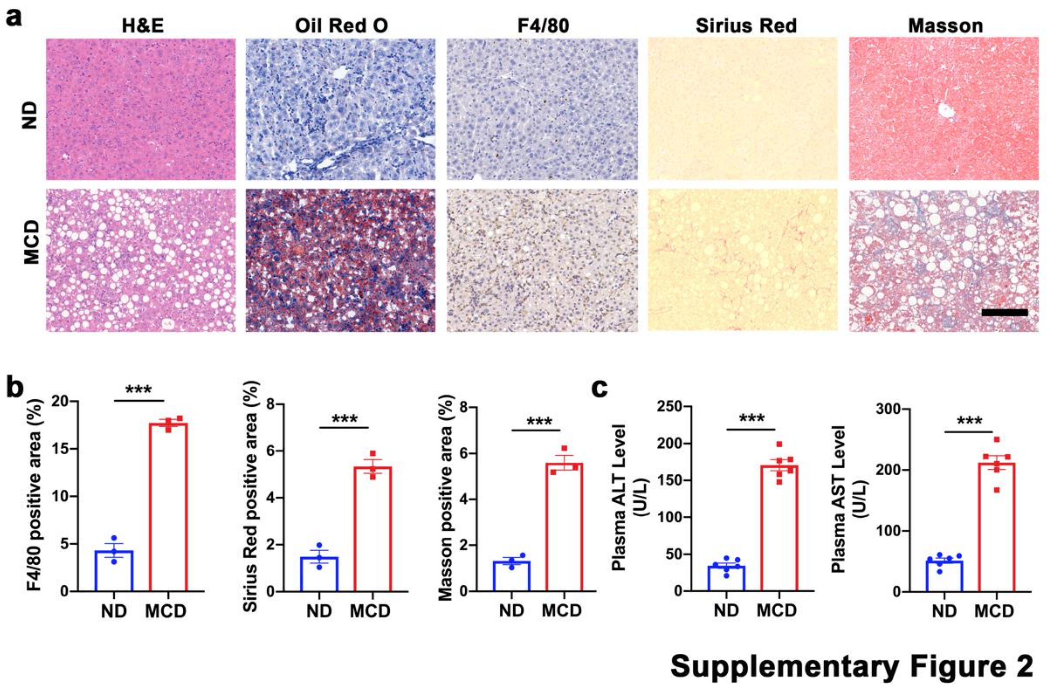

Supplementary Figure S2. Construction of MCD diet-induced NASH mice. (a-c) 8-week-old C57BL/6 male mice were fed a normal chow diet or MCD diet for 8 weeks. n=6 per group. **(a)** Liver histology analysis, including H&E, Oil red O, F4/80, Sirius red and Masson staining. Scale bar, 50 µm. **(b)** Quantitation of images. **(c)** Plasma ALT and AST levels between two groups of mice. Data are represented as mean ± SEM. *** $P < 0.001$ by 2-tailed Student's t test (b-c).

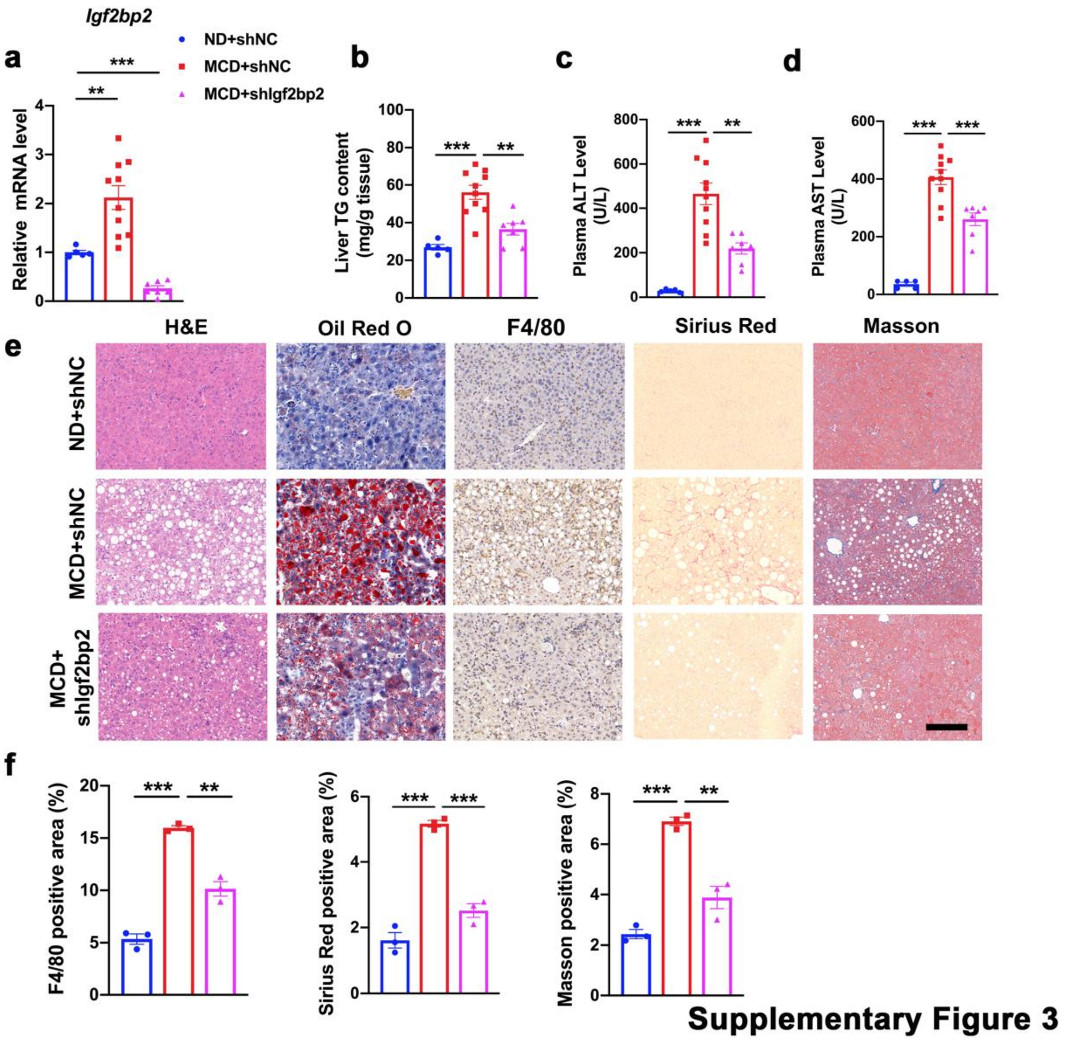

Supplementary Figure S3. Knockdown of IGF2BP2 improves hepatic steatosis, inflammation and fibrosis in MCD diet-induced NASH mice. C57BL/6J mice were fed a normal chow diet or MCD diet for 8 weeks, starting at 8 weeks of age. Then, mice were administered with adenoviral shRNA targeting Igf2bp2 or negative control for 12 days. n=5-10 per group. **(a)** Relative mRNA levels of Igf2bp2 in the livers from three groups of mice. **(b)** Liver triglyceride content. **(c-d)** Plasma ALT and AST levels. **(e)** Liver histology analysis, including H&E, Oil red O, F4/80, Sirius red and Masson staining. Scale bar, 50 µm. **(f)** Quantitation of F4/80, Sirius red and Masson staining. Data are represented as mean ± SEM. ** *P* < 0.01, *** *P* < 0.001 by 1-way ANOVA (a-d, f).

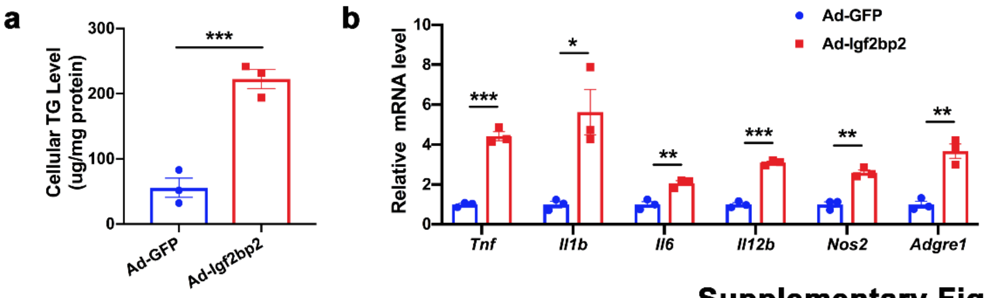

Supplementary Figure 4

Supplementary Figure S4. Effects of IGF2PB2 overexpression in mouse primary hepatocytes.

Mouse primary hepatocytes (MPH) were transfected with adenovirus containing Igf2bp2 or GFP for 24 hr. n=3 per group. **(a)** Cellular triglyceride content. **(b)** Relative mRNA levels of inflammatory genes. Data are represented as mean ± SEM. * *P* < 0.05, ** *P*<0.01, *** *P* < 0.001 by 2-tailed Student's t test (a-b).

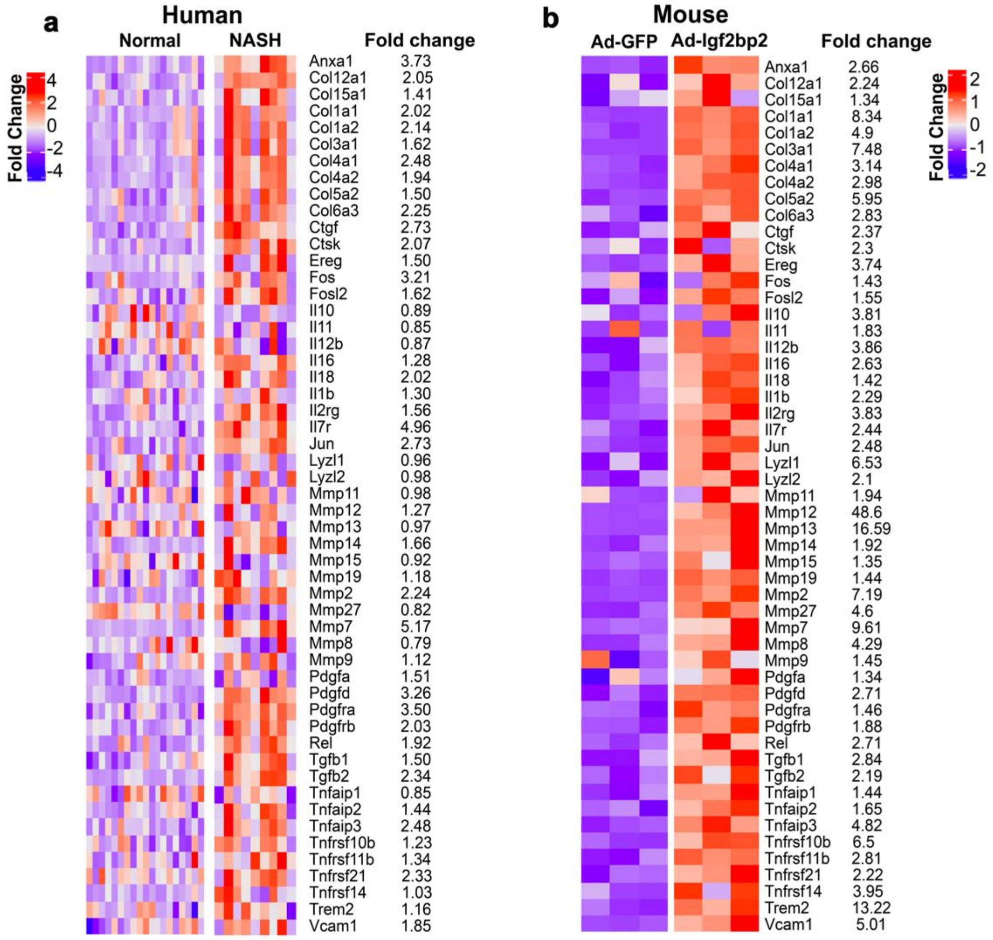

Supplementary Figure 5

Supplementary Figure S5. Comparison of gene expression profiles between human NASH livers and IGF2BP2-expressing mice.

(a) Heatmap showing the expression profile and fold change of genes involved in hepatic inflammation and fibrosis between NASH patients and normal subjects (microarray in Lake et al., Drug Metab Dispos. 2011; 39:1954-1960). **(b)** Heatmap showing the expression profile and fold change of genes involved in hepatic inflammation and fibrosis between mice injected with Ad-Igf2bp2 and Ad-GFP.

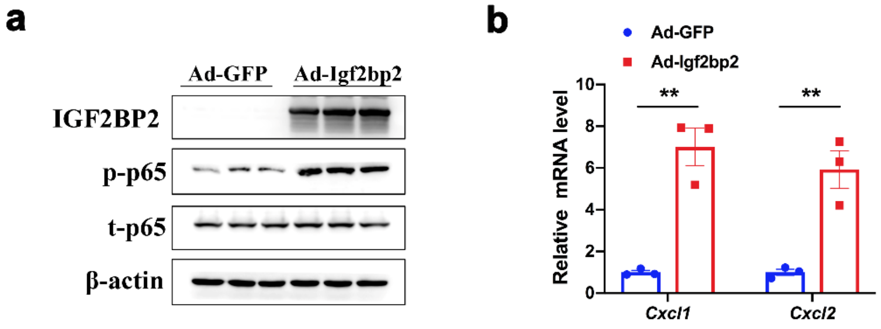

Supplementary Figure 6

Supplementary Figure S6. Activation of NF-κB signaling by IGF2PB2 overexpression in mouse primary hepatocytes.

Mouse primary hepatocytes (MPHs) were transfected with Ad-Igf2bp2 or Ad-GFP for 24 hr. n=3 per group. **(a)** Protein levels of phosphorylated p65. Total p65 were used as loading controls. p: phosphorylated. t: total. **(b)** Relative mRNA levels of Cxcl1 and Cxcl2. Data are represented as mean ± SEM. ** $P < 0.01$ by 2-tailed Student's t test (b).
